# Homogeneous Synthesis of Cationic Chitosan via New Avenue

**DOI:** 10.3390/molecules23081921

**Published:** 2018-08-01

**Authors:** Huanlu Song, Hao Wu, ShuJing Li, Huafeng Tian, YanRu Li, JianGuo Wang

**Affiliations:** 1 Beijing Advanced Innovation Center for Food Nutrition and Human Health, Beijing Technology & Business University (BTBU), Beijing 100048, China; songhl@th.btbu.edu.cn; 2 Beijing Key Lab of Plant Resource Research and Development, Beijing Technology and Business University, Beijing 100048, China; lishujing@th.btbu.edu.cn; 3 Beijing Key Laboratory of Quality Evaluation Technology for Hygiene and Safety of Plastics, Beijing Technology and Business University (BTBU), Beijing 100048, China; 4 College of Food Science and Engineering, Northwest A&F University, Yangling 712100, Shaanxi, China; wuhao2017@126.com (H.W.); 15271879994@163.com (Y.L.)

**Keywords:** chitosan, homogeneous system, quaternization, characterization, *Alicyclobacillus acidoterrestris*

## Abstract

Using a solvent formed of alkali and urea, chitosan was successfully dissolved in a new solvent via the freezing–thawing process. Subsequently, quaternized chitosan (QC) was synthesized using 3-chloro-2-hydroxypropyl trimethyl ammonium chloride (CHPTAC) as the cationic reagent under different incubation times and temperatures in a homogeneous system. QCs cannot be synthesized at temperatures above 60 °C, as gel formation will occur. The structure and properties of the prepared QC were characterized and quaternary groups were comfirmed to be successfully incorporated onto chitosan backbones. The degree of substitution (DS) ranged from 16.5% to 46.8% and the yields ranged from 32.6% to 89.7%, which can be adjusted by changing the molar ratio of the chitosan unit to CHPTAC and the reaction time. QCs inhibits the growth of *Alicyclobacillus acidoterrestris* effectively. Thus, this work offers a simple and green method of functionalizing chitosan and producing quaternized chitosan with an antibacterial effect for potential applications in the food industry.

## 1. Introduction

*Alicyclobacillus acidoterrestris* is a Gram-positive, thermos-acidophilic, non-pathogenic bacterium whose endospores are highly resistant to high temperature and acidic conditions making them survive during the pasteurization of concentrated fruit products such as commercially pasteurized apple juice, pear juice, orange juice, juice blends and canned diced tomatoes [1,2,3,4]. Undesirable odors and flavors can be produced by the bacterium as a result of guaiacol, 2,6-dibromophenol and 2,6-dichlorophenol and a light precipitation, obscure or haze in the products causes a major threat to the food industry [5,6,7]. A denser cell membrane will make the organism grow in acidic, hot media because of ω-cycloheptyl fatty acids [8].

To solve this problem, some studies have developed methods to control the microorganism in beverages. Song et al. [9] described a novel approach in which iron oxide nanoparticles (IONPs) were conjugated with the known antibiotic nisin (IONPs–nisin) to reduce the impact of *A. acidoterrestris* in fruit juices. Papain and bromelain proteases microencapsulated by alginate and chitosan exhibited inhibitory and bactericidal activity against five different species of *Alicyclobacillus* [10]. Bevilacqua et al. [11] found that the minimal inhibitory concentration (MIC) of lysozyme towards vegetative cells of the *A. acidoterrestris* varied from 0.1 to 6 ppm, while for the spores, it was from 0.1 to 3 ppm. Pei et al. [12] reported the extensive cell harm and destruction of *A. acidoterrestris* caused by paracin C, a bacteriocin produced by *Lactobacillus paracasei* CICC 20241 in apple juice. Extracts of *Piper peltatum* and *Piper marginatum* added to reconstituted orange juice were proved to be effective antibacterial agents against *A. acidoterrestris*, indicating future potential application in the food industry [4].

Chitosan (CS) can be produced from deacetylated chitin procured mainly from the exoskeleton of crustaceans [13]. Chitin is a linear polymer composed of randomly distributed units: (1→4)-2-acetamido-2-deoxy-β-d-glucan(*N*-acetyl-d-glucosamine) and (1→4)-2-amino-2-deoxy-β-d-glucan(d-glucosamine) linked by (1→4) linkages [14]. The biopolymer is considered to be chitosan when chitin is *N*-deacetylated to become soluble in dilute acidic medium [15]. Derived from the deacetylation of chitin, chitosan is a unique alkaline polysaccharide [16], which is biocompatible [17], easily biodegradable in the environment [18], and exhibits an antibacterial property [19], making it attractive for food and biomedical [20] applications.

Although chitosan has numerous applications as a stand-alone polymer or as a composite with other polymers and ceramic particles, its current use is limited. The solubility of CS in aqueous solutions is pH-dependent. The antimicrobial property of CS is poor at neutral pH as a result of the protonation of amino groups occuring only in an acidic medium [21]. Only in acidic solution less than pH 6.0 can chitosan be soluble, but further modification of CS is of interest as a strategy to obtain multipurpose materials with specific functionality as a result of the active primary amino and hydroxyl groups on the molecule [22]. A variety of derivatives produced by the modification of the CS side chain can be used for biomedical applications [23,24]. Chitosan derivatives with a quaternary-ammonium moiety are of interest, resulting in a permanent cationic charge on the polysaccharide backbone. Additionally, the poor water solubility of chitosan can be improved by the attachment of a quaternary ammonium moiety [25]. To date, a variety of polysaccharides, such as pectin, konjac glucomannan, starch, alginate, and cellulose, have been widely used with quaternization [22,26,27]. In previous reports, quaternization will improve the antibacterial ability of natural polysaccharides with a quaternary ammonium salt group. As reported, chitosan can react with methyl iodide catalyzed by sodium hydroxide to generate *N*,*N*,*N*-trimethyl chitosan (TMC) at 60 °C into *N*-methyl-2-pyrrolidinone [28]. To improve the solubility, bioadhesion, and permeation characteristics of chitosan, this two-step reductive methylation may be an attractive strategy. In another example, dissolving CS in NaOH/CHPTAC aqueous solution by use of a pressure-equalizing dropping funnel can generate *N*-(2-hydroxyl)-propyl-3-trimethylammonium chitosan chloride (HTCC) [29]. Quaternary ammonium chitosan (HACC)/polyvinyl alcohol (PVA)/polyethylene oxide (PEO) hydrogels were prepared using gamma radiation, and in vitro antibacterial activity evaluation showed that the prepared hydrogels exhibited an obvious inhibitory effect against two bacteria (*Staphylococcus aureus* and *Escherichia coli*). Other modified CSs included *N*-(2-hydroxyl) propyl-3-triethyl ammonium chitosan chloride (HTEC) and *N*-(2-hydroxyl-phenyl)-*N*,*N*-dimethyl chitosan (NHPDCS) [30,31]. The results showed that the inhibitory effects of quaternized chitosan derivatives were better than those of unmodified chitosan, and the antibacterial activities may be influenced by the cations of these modified groups. CS shows antimicrobial activity by destroying the negatively charged microbial outer membrane, and the antimicrobial activity could be improved by modifications of the CS structure [14].

In this work, a new solvent system (4.5 wt % LiOH/7 wt % KOH/8 wt % urea aqueous solution) was applied to dissolve chitosan via the freezing–thawing process to eliminate the need for conventional acid-dissolving methods of chitosan. Quaternized chitosans (QCs) were synthesized in a homogeneous system and reacted with 3-chloro-2-hydroxypropyl trimethyl ammonium chloride (CHPTAC). The structure was studied and the prepared materials were characterized. The antibacterial activity against *A. acidoterrestris* of QCs is evaluated.

## 2. Results and Discussion

### 2.1. Synthesis and Structure of Quaternized Chitosan (QC)

**Scheme 1:**
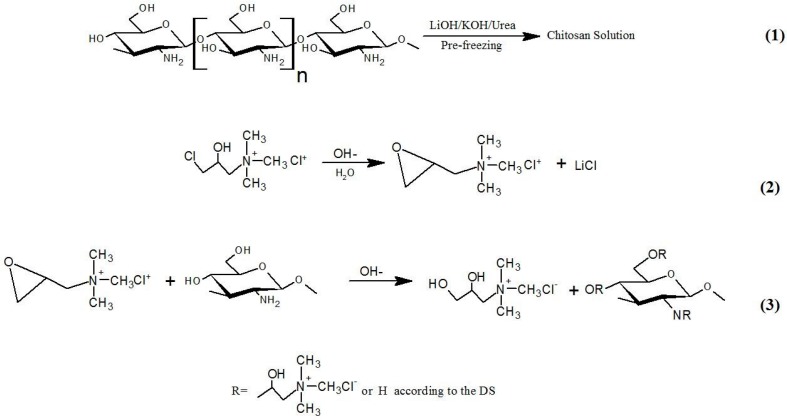
Quaternization of chitosan with 3-chloro-2-hydroxypropyl trimethyl ammonium chloride (CHPTAC) in new solvent under homogeneous system, (**1**–**3**) stand for the order of reaction.

Scheme 1 shows the one-pot synthesis strategy for quaternized chiosan. Initially, 2 g chitosan powder was dispersed in an environment-friendly LiOH/KOH/urea/H_2_O solution at a ratio of 4.5%:7%:8%:80.5% by weight and then stirred slowly for 3 min using mechanical agitation, aiming to swell the chitosan sufficiently. The turbid liquid solution of chitosan was stored under refrigeration at −30 °C overnight. The frozen solid was then fully thawed and stirred extensively by mechanical stirring at room temperature. To modify the amino groups of chitosan, CHPTAC was used as an etherification reagent and formed epoxide under alkaline conditions and reacted with the sodium alkoxide of chitosan.

The reaction conditions for the synthesis of quaternized chitosan are summarized in Table 1. During the course of the reaction, CHPTAC decreased slowly to maintain the solution transparency and remain completely homogenous. After dialysis in distilled water and lyophylization, the DS ranged from 16.5% to 46.8% and the yields varied from 32.6% to 89.7% by varying the reaction temperature, the molar ratio of CHPTAC to chitosan unit, and the reaction period. The quaternized chitosan samples were coded as QC1–QC7, according to their synthesis conditions (as shown in Table 1). The zeta potential of QCs increased from +45.8 mV to +54.2 mV with increasing DS, which could be attributed to the introduction of a higher amount of quaternary ammonium salts onto the chitosan backbones [32].

Under room temperature (25 °C) conditions, the QC products were synthesized easily and showed good water solubility and high degree of substitution (DS). The transmittances of the solutions were 92.3%, 82.5%, 84.0%, 82.1%, 80.4%, 54.9%, 3.7%, respectively. We defined transmittance above 80% as “++” and transmittance above 50% as “+”, while QC7 was insoluble. With a higher reaction temperature of 40 °C, QC-6 were obtained with poor water solubility, lower DS, and yield. When the temperature was above 40 °C, the quaternized reaction was generally difficult to process. For QC-7, under a reaction temperature of 60 °C, very little sample was obtained, likely due to the gel formation of chitosan solution at high temperature. The conductivity titration revealed that with an increasing molar ratio of CHPTAC to chitosan unit and reaction time, the DS of QCs increased while the DS of QCs decreased with the increasing of reaction temperature. Fortunately, the homogeneous synthesis of QCs could be conducted at temperatures below 40 °C, which avoided rigorous reaction conditions and high cost.

### 2.2. Fourier-Transform Infrared (FTIR) Spectrometry Analysis

**Figure 1:**
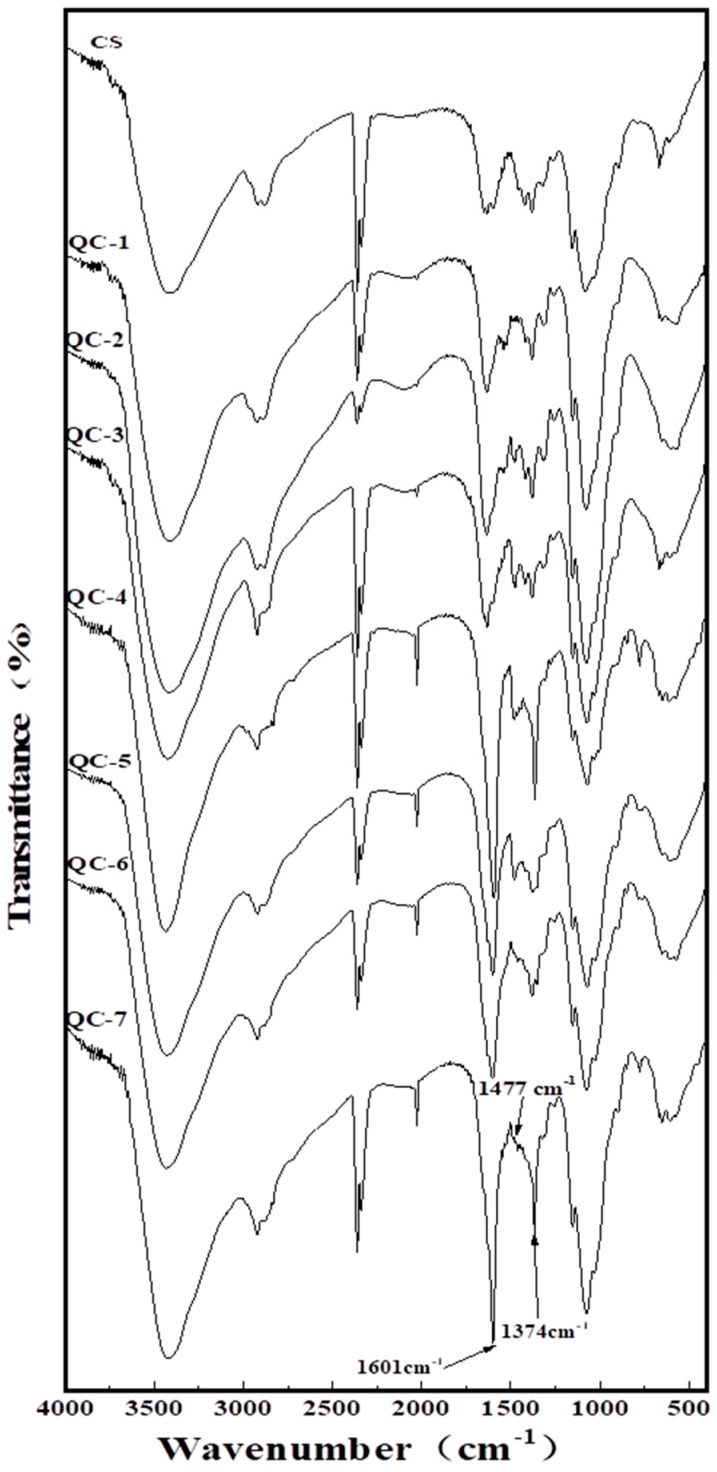
Fourier-transform infrared (FTIR) spectra of chitosan and QCs.

The Fourier-transform infrared (FTIR) spectra of chitosan and QCs are shown in Figure 1. Absorption peaks at 1650 cm^−1^ and 1590 cm^−1^ were assigned to C=O stretching (Amide I) and N–H bending (Amide II) of the glucosamine unit, respectively. After the reaction, there was some decrease in the peak intensity of the N–H bending of chitosan at 1590 cm^−1^ [33]. The band of the salt positioned at 1374 cm^−1^ and 1477 cm^−1^ is the most striking difference between the two spectra, which corresponds to an asymmetric angular bending of the methyl groups of quaternary hydrogen, indicating substitution of the alkyl groups occurred at the amino groups of chitosan. These peaks were not detected in the infrared spectrum of the original chitosan. The N–H bending (1601 cm^−1^) of the primary amine was weak due to the change of the primary amine to the secondary amine [34], and a broad peak in the range of 3300–3500 cm^−1^ was due to the vibration of the hydrogen bonded –NH_2_ and –OH [35].

### 2.3. ^1^H Nuclear Magnetic Resonance (NMR) Characterization of QCs

**Figure 2:**
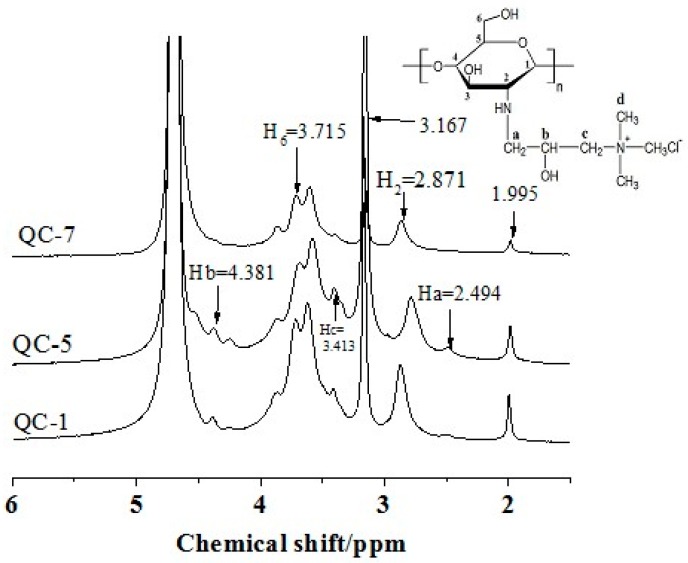
^1^H NMR of QC-1, QC-5 and QC-7.

The ^1^H nuclear magnetic resonance (NMR) spectra and the sketched structure of QC-1, QC-5, and QC-7 are shown in Figure 2, where the protons of QCs are coded as H-1 to H-6, and H-a to H-d, separately. A very strong peak at around 3.167 ppm was observed, sugguesting the presence of methyl groups (H-d) in the quaternary ammonium side chains. Peaks at 2.494, 4.381, and 3.413 ppm were ascribed to H-a, H-b, and H-c. For QC-7, we can conclude that peaks for H-a, H-b and H-c that were barely detected indicated the low DS when the reaction temperature was 60 °C, which is in line with the results of FTIR and DS analysis by using the conductivity titration apparatus. The remaining peaks, assigned to protons in the chitosan backbone and in quaternized side chains, were well in compliance with reported results [36].

Based on the IR and ^1^H NMR spectra of QCs, conclusions can be drawn that quaternary ammonium side chains were successfully grafted onto the chitosan main chains, and quaternized chitosan was successfully synthesized in this new homogeneous aqueous solution, where the quaternary ammonium salt group could be introduced onto chitosan chains under alkali conditions.

### 2.4. X-ray Diffraction (XRD) Analysis of QCs

**Figure 3:**
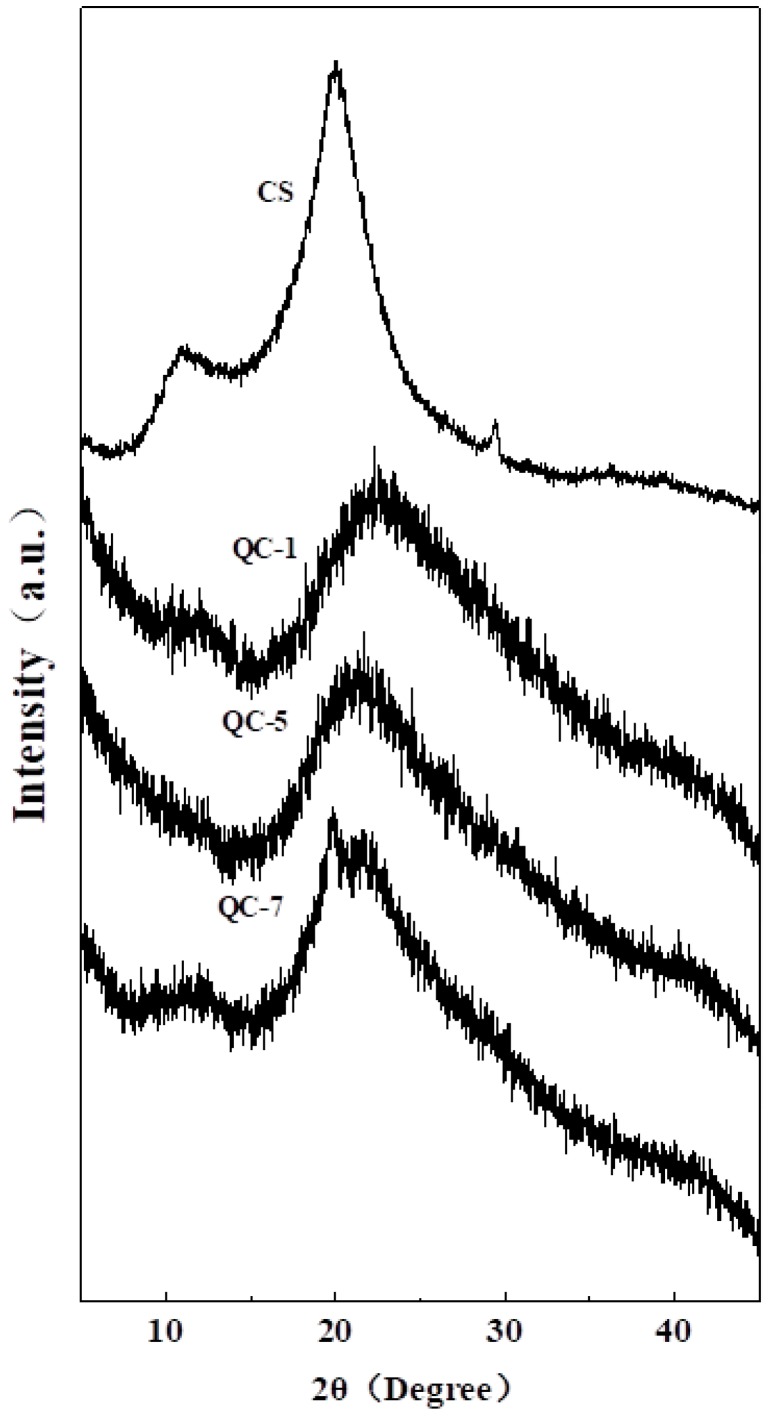
X-ray diffraction (XRD) patterns of chitosan and QCs.

Figure 3 shows the X-ray diffraction patterns of chitosan and QCs with different DS. For chitosan, one intense characteristic peak was observed at 2θ of 20° in the XRD pattern, which is the characteristic peak for chitosan. Meanwhile, two peaks at 2θ of 11° and 29° were due to the strong hydrogen bonding of chitosan, suggesting the well-organized arrangement of chitosan molecules and poor water solubility [37]. In the X-ray diffraction patterns of QC-1 and QC-5, the peaks at 2θ of 11°, 20° and 29° were barely observed. Instead, a new broadened peak at 22° was detected. For QC-7, weak peaks at 2θ of 11° and a strong peak at 2θ of 20° were still detected, revealing the insufficient reaction of chitosan at 60 °C [38]. All the results suggested that the crystal structure of QCs is greatly affected by grafting quaternary ammonium salt groups onto the chain of chitosan, and the reduction of the number of free amino groups and hydroxyl groups in the QCs leads to a decreased hydrogen bonds between the molecules and the molecular chain. The attachment of quaternary ammonium groups destroyed the original regularity of the chitosan molecules, but at the same time the water solubility of QCs was significantly enhanced [39].

### 2.5. Differential Scanning Calorimetry (DSC) Analysis of QCs

**Figure 4:**
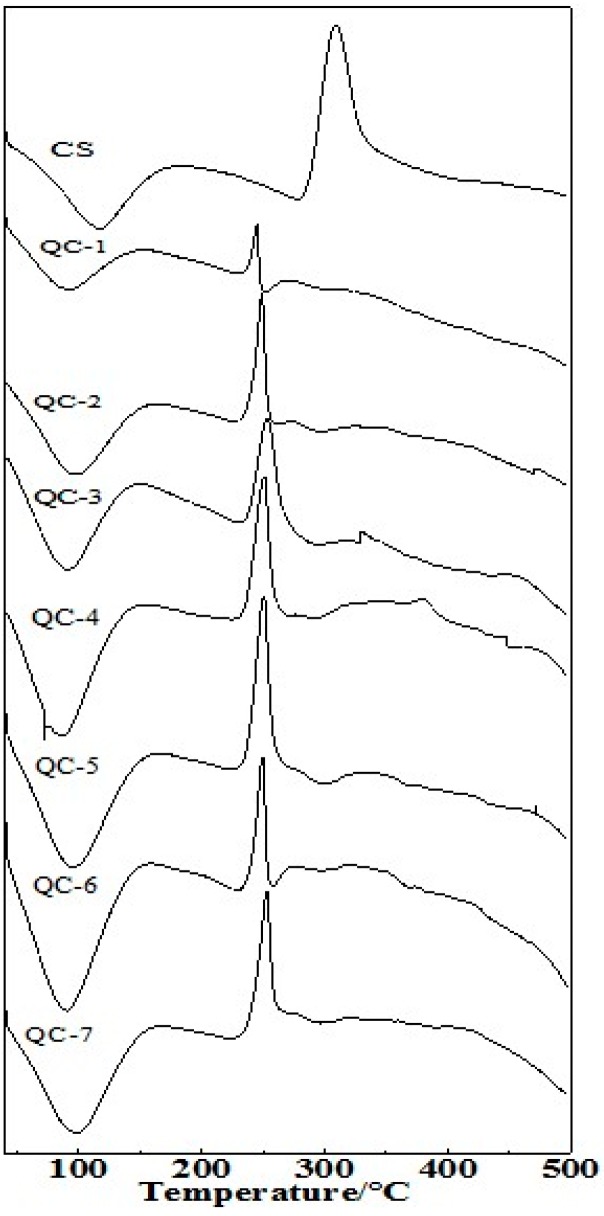
DSC thermograms of chitosan and QCs.

The thermal behavior of all samples was investigated using differential scanning calorimetry (DSC) under nitrogen atmosphere, as shown in Figure 4. Native CS exhibited a broad endothermic peak at 116.8 °C and an exothermic peak at 308.1 °C, which was atttributed to the loss of bound water and the decomposition of the CS backbone, respectively [40,41,42]. The endothermic peak of QCs, were in the range of 72.0–98.5 °C with an onset at 41.7–51.0 °C (Table 2). Basically, polysaccharides can be disordered to be hydrated as they have a strong affinity with water [43]. Therefore, the endotherm associated with water evaporation is expected to reflect physical and molecular changes during the introduction of the quaternary ammonium moieties into the CS backbone. This can be observed in the difference in peak area and endothermic peak position, indicating that the water holding capacity of these CS derivative backbones, the strength of water and the CS derivative skeleton interaction are different [44,45]. In Table 2, the CS has an enthalpy ΔH of 230.1 J/g, but the QCs have an ΔH value that ranged from 277.2 J/g to 347.5 J/g. This indicates that introduction of the quaternary ammonium moiety into the CS backbone results in an increase in water retention capacity. Similarly, the ΔH of QC increases as DS increases.. It is noteworthy that QC-5 exhibited the highest enthalpy ΔH value, 347.5 J/g, caused by the highest degree of substitution and strongest water holding capacities. These results were confirmed by the X-ray diffraction pattern of the QCs.

The exothermic peak at 308.1 °C was attributed to the decomposition of the CS backbone, and the exothermic peak of the QCs shifted to lower temperature from 245.0 to 253.7 °C. The result revealed a decrease in thermal stability as a result of decreased crystallinity, which is attributed to an introduction of the quaternary ammonium moiety into the CS backbone. The exotherm and endotherm at lower temperatures can be ascribed to the decomposition of highly crystal regions in the CS backbone [46].

### 2.6. Antibacterial Activity

**Figure 5:**
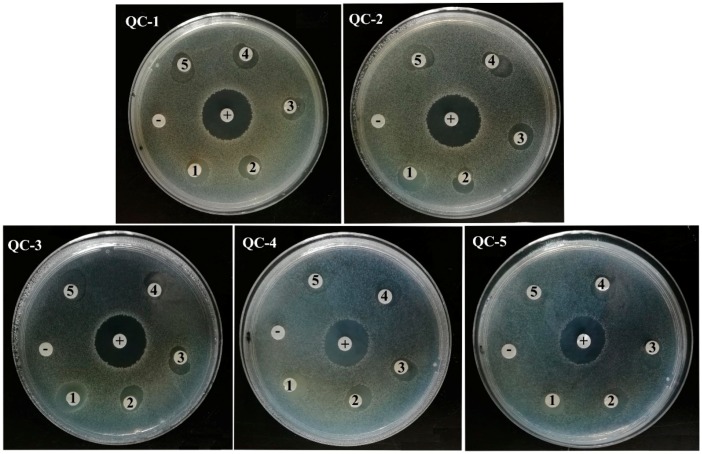
Antimicrobial activities against *Alicyclobacillus acidoterrestris* DSM 3922^T^ of QCs. (“+”: 100 μg/mL Nisin in pH = 5 HCl aqueous solution, “−”: control group of pH = 5 HCl aqueous solution, “1”: 5 mg/mL QC solution in pH = 5 HCl aqueous solution, “2”: 2.5 mg/mL QC solution in pH = 5 HCl aqueous solution, “3”: 1.25 mg/mL QC solution in pH = 5 HCl aqueous solution, “4”: 0.625 mg/mL QC solution in pH = 5 HCl aqueous solution, “5”: 0.3125 mg/mL QC solution in pH = 5 HCl aqueous solution.

Figure 5 shows the inhibition zone of the samples. The diameters of the inhibition zones are shown in Table A1. When the concentration of samples reached 0.3125 mg/mL, a clear transparent zone of inhibition could be observed. However, the diameter of the zone of inhibition showed no obvious change with the increase of the samples’ concentration and this was true for all samples. This may be due to the twine and enfoldment between large molecules of the QCs and the agar, and poor solubility of QCs which leads to weak diffusion of QCs on the plates [47]. This means that some methods of direct full contact between test bacteria and samples should be considered. The MIC values for QC-1 to QC-5 were 500, 400, 250, 100 and 75 μg/mL, respectively. With the increase of the degrees of substitution of the sample and the zeta potential, the MIC values gradually decrease, indicating that the antibacterial ability is enhanced. This may be attributed to the interaction of gradual increased positive charge on the sample molecules with negatively charged bacterial surfaces, which results in loss of cell membrane permeability, seepage of cell contents, and eventually bacterial death [48].

## 3. Materials and Methods

### 3.1. Materials

Chitosan (degree of deacetylation of 85%; viscosity of 1% chitosan solution at 20 °C at 1250 mPa·s; *M_w_*: 2.39 × 10^5^ Da by dynamic light scattering) was purchased from Aladdin Reagent Co. (Shanghai, China). The degree of deacetylation (*DD* = 89%) of CS was determined by the two-abrupt-change potentiometric titration method and calculated using the following equation:

(1) $$\alpha =\frac{\Delta V\times C_{NaOH}\times 10^{-3}\times 16}{m\times 0.0994}\times 100\%$$

where *ΔV* and *C_NaOH_* stand for the volume and concentration of NaOH consumption between the two abrupt changes of pH, respectively, *m* is the dry weight of a chitosan sample, and *α* is the *DD* of the chitosan sample. 3-chloro-2-hydroxypropyltrimethylammonium chloride (60 wt % in water) was purchased from Aladdin Reagent Co. (Shanghai, China) and was used as an etherifying reagent without further purification. Urea and sodium hydroxide (Analytical reagent, AR) were purchased from Hua Da Reagent Co. (Guangdong, China). Nisin was purchased from Sigma Aldrich (Shanghai, China). Distilled water (electrical resistance ≈ 18.2 MΩ·cm) was used to prepare all aqueous solutions. Other chemical reagents were obtained from commercial sources in China and were of analytical grade and used without further purification.

### 3.2. Homogeneous Synthesis of QC

Alkaline aqueous solution containing LiOH/KOH/urea/H_2_O at a ratio of 4.5%:7%:8%:80.5% by weight was used to solve chitosan. To prepare the solutions, 2 g chitosan powders were dispersed into the alkaline aqueous solvent with stirring for 3 min and then the solutions were stored at −30 °C overnight. Then, the frozen solid was fully thawed and stirred extensively by mechanical stirring at room temperature. During the stirring, the “Weissenberg Effect” was obviously observed. After centrifugation at 7000 rpm for 10 min at −5 °C to remove air bubbles, a transparent chitosan solution at a concentration of 2 wt % was obtained [16], indicating the successful preparation of the homogeneous chitosan solution. To synthesize QCs, a certain amount of CHPTAC aqueous solution was added dropwise to the chitosan solution and the resulting mixture was stirred for a predetermined period at the required temperature. Then, the reaction solution was neutralized by the addition of 1M HCl aqueous solution and then dialyzed against distilled water for 7 days (*M_w_* = 8000 cut-off, Union Carbide Corporation, Danbury, CT, USA). Finally, white QC powder was obtained after lyophilizing.

### 3.3. Estimation of Water Solubility

The water solubility of QCs at aqueous solution was determined by turbidity measurement [46], in which an aqueous solution of quaternized chitosan (5 mg/mL) was prepared by dissolving quaternized chitosan in deionized water with magnetic stirring overnight at room temperature. The transmittance of the solutions was measured using a UNICO UV-2000 Spectrophotometer (UNICO Inc., Franksville, WI, USA) at 600 nm.

### 3.4. Characterization of QC

The FTIR of QCs were determined by the method of KBr pellets on a Nicolet 5700 Fourier transform infrared spectrometer Acros (Thermo Electron Scientific Instruments Corp., Waltham, MA, USA).

### 3.5. Degree of Substitution (DS) of QCs

Using a conductivity titration apparatus, the degree of substitution (DS) of QCs was determined by titrating the chloride ions with AgNO_3_ solution and then calculation using the following equation [49]:

(2) $$DS=\frac{V\times c/1000}{V\times c/1000+(w_{1}-V\times c\times 314/1000)/162}\times \frac{1}{DD}\times 100\%$$

where *V* (mL) is the volume of the AgNO_3_ solution, *c* (mol/mL) is the concentration of AgNO_3_ solution, and *w*_1_ (g) is the weight of QCs (314 is the molecular weight of the repeated unit of quarternized chitosan and 162 is the molecular weight of the repeated unit of chitosan). *DD* is the degree of deacetylation of chitosan.

### 3.6. Zeta Potentials of QC

The zeta potentials of QCs were measured on a Nano-ZS ZEN3600 (Malvern Instruments, Malvern, UK) at 25 °C. Before measurement, samples was dissolved in distilled water to prepare a test solution (1 mg/mL) and then filtered using millipore filter (0.22 μm).

### 3.7. Nuclear Magnetic Resonance (NMR) Characterization

^1^H nuclear magnetic resonance (^1^H NMR) was operated on a NMR spectrometer (AVANCEIII-600, Bruker) (Karlsruhe, Germany). A certain amount of QCs was dissolved in D_2_O to prepare a 5 wt % solution. Chemical shifts were given in ppm and tetramethylsilane (TMS) was used as an internal reference.

### 3.8. X-ray Diffraction (XRD)

XRD tests were carried out on an XRD diffractometer (D8 ADVANCE A25, Bruker) (Karlsruhe, Germany). The XRD patterns with Cu *K_α_* radiation (*λ* = 0.154 nm) at 40 kV and 50 mA were recorded in the region of 2θ from 5° to 40°.

### 3.9. Differential Scanning Calorimetry (DSC)

The thermo stabilities of QCs were determined by DSC (Waters, Q2000) (Worcester County, MA, USA). Approximately 3 mg of sample was weighted in hermetic pans and an empty hermetic pan was used as control. QCs were heated from 25 °C to 250 °C at 10 °C/min.

### 3.10. Antibacterial Activity

The antibacterial activities of QCs against *Alicyclobacillus acidoterrestris* DSM 3922^T^ were evaluated by the filter paper method [50] with slight modification. In short, the strain was propagated in the *Alicyclobacillus* spp. medium (AAM) and incubated following the method reported [9]. QCs were added to HCl solution (pH = 5) to make 5 mg/mL stock solutions that were stirred overnight and diluted to 2.5, 1.25, 0.625, and 0.3125 mg/mL using the 2-fold dilution method, respectively. Nisin was dissolved in the HCl solution as positive control (0.1 mg/mL) while blank HCl solution was used as negative control. The filter paper pieces with a diameter of 6 mm were immersed in gradient dilutions of each sample and soaked for 1 h and then attached to the AAM agar (2% agar) plates mixed with approximately 10^6^ CFU/mL freshly propagated strain of *A. acidoterrestris*. The samples were incubated for 24 h at 45 °C and the zone of inhibition was measured to evaluate the antimicrobial activity.

The minimal inhibitory concentration (MIC) of QCs was investigated by microdilution in 96-well microplates in AAM broth according to an earlier method [51] with slight modification. Each well contained 20 μL suspension of test bacteria (2.5 × 10^4^ CFU/mL), 5 μL of serially diluted samples (500, 400, 250, 200, 150, 100, 75, 50 μg/mL) and 175 μL AAM broth. The positive and negative control were the same with above filter paper method, respectively. The plates were checked by sight to detect if the growth of the bacteria is inhibited after being incubated at 45 °C for 24 h. The MIC was defined as the lowest concentration of each test sample where no opacity resulting from bacterial growth could be observed.

## 4. Conclusions

The purpose of this work was to develop a green and novel approach to synthesize quaternized chitosan in a homogeneous system. Using a frozen/thawing solution containing LiOH/KOH/urea/H_2_O at a certain weight ratio, chitosan can become totally dissolved if the “Weissenberg Effect” is observed when the chitosan solution was stirred with mechanical agitation. The quaternized chitosan can be obtained easily when the reaction temperature was lower than 40 °C in a homogeneous solution, supported by the results of FTIR and ^1^H NMR. The strong intramolecular and intermolecular hydrogen bonds and crystalline region of chitosan were destroyed after quaternization, which may explain the improved water solubility. The innovative method described herein may provide an environmentally friendly strategy to prepare QCs soluble in water. QCs exhibit a good antibacterial activity against *Alicyclobacillus acidoterrestris* DSM 3922^T^. This paper may present significant applications in food fields such as food hydrocolloids, the flocculabilities, heavy metal removal, and anti-microbial activities. Exploration of these important applications is required.

## Figures and Tables

**Table 1:** The reaction conditions, yields, degree of substitution (DS), solubility and zeta potentials of quaternized chitosans (QCs).

Sample	Volume of CHPTAC	React Time (h)	React Temperature (°C)	Yield (%)	Degree of Substitution (%)	Solubility	Zeta Potentials (mv)
QC1	6	15	25	68.9	32.9	++	45.8 ± 1.1
QC2	12	15	25	78.6	36.1	++	48.1 ± 1.5
QC3	24	15	25	85.2	44.5	++	49.6 ± 0.5
QC4	24	30	25	89.7	46.6	++	53.8 ± 1.9
QC5	24	45	25	88.5	46.8	++	54.2 ± 0.5
QC6	6	15	40	48.7	25.3	+	-
QC7	6	15	60	32.6	16.5	-	-

**Table 2:** Peak temperature in DSC during the thermal degradation and water evaporation of chitosan (CS), quaternized chitosan (QCs). (The line break of Table 2 stands for the “endothermic” and “exothermic”, seperately).

Sample	Onset/°C	Peak/°C	Terminal/°C	DH (J/g)
QC-1	51.7 238.4	89.6 245.0	152.4 252.6	284.8 −73.3
QC-2	47.4 240.4	96.2 249.0	160.6 262.5	298.1 −71.8
QC-3	45.0 238.5	90.4 253.7	150.4 293.0	277.2 −108.6
QC-4	41.7 236.7	72.0 251.2	151.9 274.2	302.5 −89.1
QC-5	44.5 237.3	94.0 250.8	148.9 293.9	347.5 −121.0
QC-6	48.4 240.5	93.6 249.8	136.5 258.4	285.2 −38.9
QC-7	51.0 241.6	98.5 253.1	153.1 296.2	280.9 −92.1
CS	64.7 288.1	116.8 308.1	179.3 338.4	230.1 −196.4

## Appendix A

**Table A1:** Antibacterial activities of QCs.

Dilution Gradient	Diameter of Inhibition Zone (mm)				
	QC-1	QC-2	QC-3	QC-4	QC-5
1	11.64	13.32	15.96	12.44	11.66
2	10.50	12.30	12.28	13.46	12.46
3	10.76	12.54	12.30	12.22	13.06
4	12.02	12.94	12.14	12.46	12.02
5	11.98	13.36	13.86	13.26	12.22
positive control	23.80	24.90	23.84	21.76	22.34
negative control	11.64	13.32	15.96	12.44	11.66
